# Characterization of Interface Transition Zone in Asphalt Mixture Using Mechanical and Microscopic Methods

**DOI:** 10.3390/ma17215197

**Published:** 2024-10-25

**Authors:** Mujaheed Yunusa, Wenqi Hou, Guoqing Jing, Hao Wu

**Affiliations:** 1 School of Civil Engineering, Central South University, Changsha 410075, China; 2 Mechanical Teaching and Experimental Center, Central South University, Changsha 410075, China; 3 School of Civil Engineering, Beijing Jiaotong University, Beijing 100044, China

**Keywords:** hot recycled asphalt mixture, rejuvenator, interface transition zone, microscopy, mechanical properties

## Abstract

An enormous surge in the pavement sector requires the evaluation of interface bonding in asphalt composite, since the assessment of bonding brings considerable cost savings. Microscopic and mechanical analyses were performed to study the status of the interface transition zone of four groups of asphalt mixtures, using thin-slice preparation to obtain asphalt mixture slices with a flat surface for microscopic analysis. The interface transition zones were characterized using good knowledge of blending or diffusion phenomena by conducting tests both at the micro and macro levels to determine mixture quality. Asphalt mixture components were observed using fluorescence microscopy imaging and evaluated by the gray value change law. Asphalt mixture groups, (virgin, recycled of 30% aged and 70% unaged, 6%, and 4% rejuvenator dosage mixtures) under the same process parameters, which are a mixing time of 270 s and a mixing temperature of 150 °C, been considered optimum for component fusion in a hot asphalt mixture were used. This study relied on the influence of morphology law, assessed through rutting tests for high temperature performance, semi-circular bending tests for low temperature performance, and pull-off tests for interface bonding strength. The relationship between interface transition zones and macro performance was studied. The self-developed pull-off method was a research innovation which can be used as an alternative to study interface transition zones in asphalt mixtures, and provides the necessary data needed with 3D surface failure mode calculations. The device measured the bonding strength of a single aggregate in distinct positions using the bitumen penetration test method. The main goals were to determine a correction factor, identify the appropriate alteration, and compute the actual fracture surface area. Using scanning electron microscopy for interface characterization and micro-morphologies of mortar transition zone, our analysis provides adequate knowledge about interface position and the components present. The applied approaches to characterize asphalt mixture interfaces proved workable and reliable, as all methods have similar trends with useful information to determine asphalt pavement quality.

## 1. Introduction

In terms of impacting the pavement’s overall performance, the interface transition zone (ITZ) between the paste matrix and particles is often considered crucial. For quite some time, it has been an important focus of pavement engineering [1]. It is acknowledged that the ITZ is influenced by the “wall effect”, wherein the binder’s packing is interrupted by the surface of the aggregates [2]. The ITZ influences pavement performance but is mostly overlooked when designing an asphalt mixture. The region is a micro zone at the aggregate surface that demonstrates a notable change in mechanical performance. The ITZ between aggregate surface and asphalt mortar is regarded as a sensitive region that influences asphalt mixture performance [3,4,5]. Interface failure between the aggregate and asphalt mortar typically happens during vehicle loading, on account of its weak adhesive strength and the complex stress conditions. Therefore, the ITZ is generally viewed as the “weak link” in asphalt mixtures, and it comparatively high porosity offers an easier conduit for aggressive ions to enter into the asphalt mixture. Thus, examining the mechanical properties and micromorphology of the ITZ is vital to figure out the fracture process of the interface zone. It is also useful for successful damage assessment and prediction. Yet, existing technology cannot successfully measure and analyze the interface zone due to its microscale size. Nanomechanical properties and microstructures of the interface zone were proposed based on nanoindentation and SEM research to better understand its morphology and mechanical behavior [6]. With many accessible small-volume specimens, the technique may efficiently evaluate the basic properties of distinct phases and crucial spots in heterogeneous mixtures [4].

Moreover, the relationships between ITZ’s attributes and the transport qualities of asphalt mixtures have been explored by numerous researchers [7]. Wong et al. [8] assessed the impact of ITZ and microcracking on the transport characteristics of cement-based materials after drying. It was shown that the net influence of ITZ on overall transport features is less substantial, yet the effects of total porosity, tortuosity, and small cracks on transport characteristics, notably, permeability, are considerably more significant [9]. G. Fang et al. [10] investigated the mechanism of microstructure assessment of ITZ in alkali-activated fly ash-slag concrete using a scanning electron microscope (SEM). (SEM), X-ray diffraction (XRD), atomic force microscopy (AFM), and nanoindentation are used to detect the characteristics of microstructures, mineral compositions, and interface zones [11,12]. Aggregate and asphalt mortar, in general, modify the mechanical behavior of interface zones, which are commonly cracks due to stress concentration and adhesion failure.

Studying the internal morphology and structure of asphalt mixtures is critical for enhancing the service performance of asphalt pavements [13,14]. Various studies have been conducted on the internal properties of asphalt mixtures, and several research findings have been acquired [15]. Wang Feng et al. [16] quantified aggregate skeletal structure, including aggregate orientation, dispersion, and contact qualities, using a digital image processing approach. Xiao et al. [17] analyzed the micro-dispersion morphology of the styrene butadiene styrene (SBS) modifier using a Nikon fluorescence microscopy device with thin-section preparation methods. Fluorescence technology was used to investigate SBS morphologies in asphalt and determined that SBS morphologies and viscoelastic binder qualities are related [18].

Asphalt pavement recycling is gaining popularity worldwide [19,20,21,22]; in road construction, two methods are often used: hot recycling and cold recycling of reclaimed asphalt pavement (RAP) [23,24]. Environmental aging will occur during pavement service life, causing components of the asphalt to volatilize or change, resulting in cohesive fracture or interface failure of the asphalt mixture due to asphalt embrittlement and adhesion loss [25,26,27,28]. Because the reclaim asphalt (RA) has stiffened due to aging in the pavement throughout its service life, hot mixture asphalt recycling typically requires adding a softer asphalt or a rejuvenator during the mixing process to soften the stiffer RA. Blending charts or equations such as the logpen rule are often used to calculate the grade of additional soft asphalt in recycled asphalt mixtures, assuming that all RAs are homogeneously and thoroughly mixed with virgin asphalt in the mixing process [5]. Several laboratory and field experiments have revealed that the latter assumption is usually entirely correct [29]. A non-homogeneous, partial mixing of RA with soft virgin asphalt may reduce fatigue life [30]. Blending degree has been determined using two separate approaches: the so-called mechanistic and interface detection techniques. Mechanistic technique attempts to quantify the amount of blending indirectly by comparing the rheological characteristics of RA mixtures with ideal mixes in which the extracted RA has already been mixed with virgin asphalt before mixing [31,32]. Blending measurements in interface detection techniques are mostly based on investigating diffusion between two binders or between binder and rejuvenator. Recycling and rejuvenation of these materials are becoming more critical in the global pavement industry [33,34]. Xiao et al. [35] improved the performance of reclaimed asphalt pavement by adding waste rubber to it.

### Problem Statement and the Objectives of the Study

Recently, researchers have found that the interaction between asphalt mixture constituents determines asphalt road durability [36]. According to several studies, the interface zone plays a crucial role in the occurrence of distresses, particularly in recycled or aged mixture [37]. The blending degree of aged and virgin asphalt components in a hot recycled mixture and the diffusion of rejuvenators in an aged asphalt mixture are crucial to interface adhesion, especially when considering the parameters that asphalt mixture undergoes during production time or service period. Previous studies focused on determining the optimum percentages of aged to virgin mixture [38] and suitable additives while neglecting the power of interface bonding or solubility in determining the structural and physical properties that the mixture undergoes. This article’s focus was solely on interface evaluation using unique and simple methods with consideration of the parameters that an asphalt mixture undergoes, such as analysis of the extent of miscibility, rejuvenator dosage, and interface failure. It will provide useful approaches and new understanding regarding interface study at the micro and macro level and the relationship between the interface zone and asphalt mixture performance.

This study will rely on a set of interface region characterization methods in asphalt mixtures, including microscopic morphology of the ITZ, and interface failure with three-dimensional (3D) fracture surface calculation. The study’s principal objectives are:

➢ Microscopic analysis to evaluate the status of ITZ in asphalt mixtures and its relationship with mechanical performance.
➢ Interface strength study using a pull-off test with a 3D failure surface area acquisition to ensure a better failure strength calculation, and a semi-circular bending (SCB) test for low-temperature fracture resistance while maintaining dynamic stability for high temperature performance. It allows us to determine the possible correction factor for the entire fracture area and examine how the ideal test modification affects the fracture surface. This research demonstrates that it is possible to compute correction factors for various asphalt mixtures.

## 2. Experimental Program

**Figure 1:**
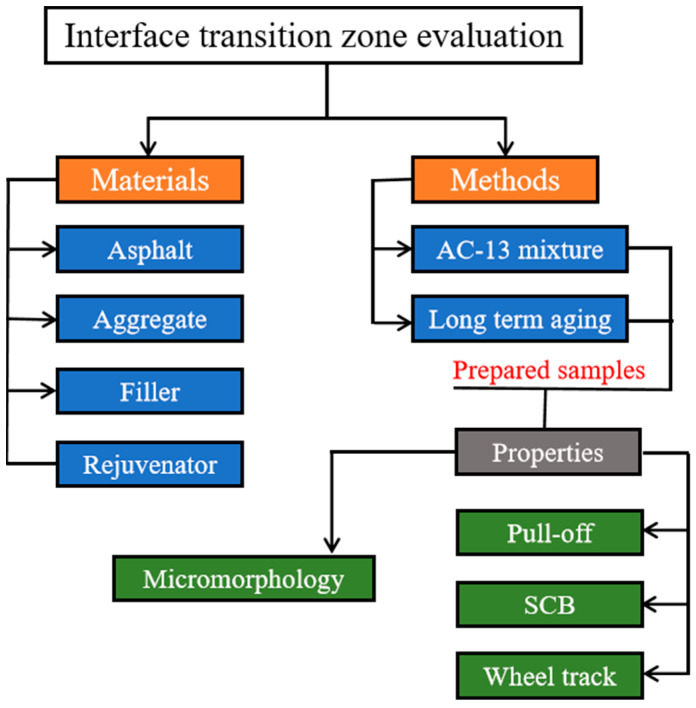
Procedure of the research.

Figure 1 displays the procedure of the research. After materials acquisition, long-term aging of the recycled mixture preparation was considered and conducted in the laboratory using AASHTO R 30 procedures [39]. Loose asphalt mixtures were distributed to a depth of 50 mm on aging trays and then placed in a convection oven at 85 °C for 120 h for the RAP preparation. To achieve homogeneity during the aging process, the asphalt mixtures were stirred every 8 h. Several studies were performed to optimize this method, and it was discovered that the binder derived from such processes is of the same extent as long-term aging asphalt [40]. The recycled asphalt mixture’s proportions were 30% aged and 70% unaged. Tests for various characterization sample preparations were made with full details in the subsections.

### 2.1. Materials

**Figure 2:**
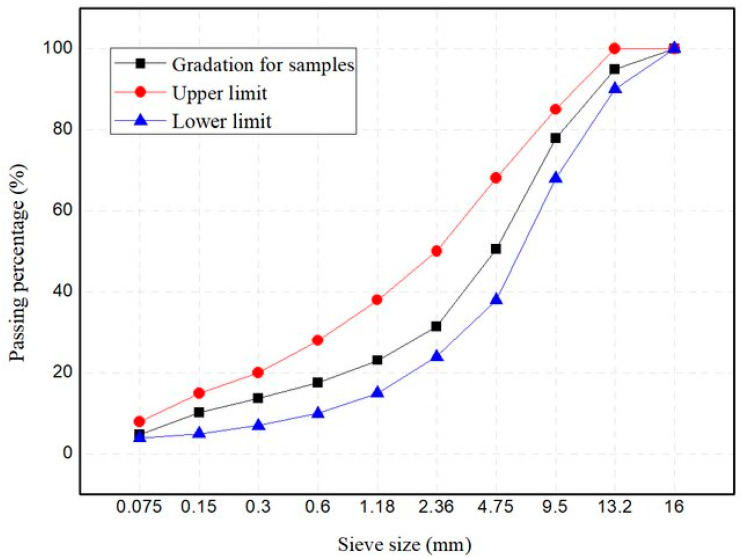
Aggregate gradation of the AC-13 asphalt mixture.

AH-90 asphalt binder, basalt aggregate, limestone mineral filler, and commercial rejuvenator were used in this study; their basic properties are listed in a tabular form. Table 1 presents the physical properties of basalt aggregate, a typical road-building material used in asphalt mixtures. The aggregate gradation design of AC-13 with an ideal binder ratio of 4.9 wt% is shown in Figure 2. The physical parameters of the asphalt binder employed are shown in Table 2; this type of pliable asphalt binder is more suited for areas with lower temperatures. The parameters of limestone chosen as a mineral filler are shown in Table 3. To rejuvenate the aged asphalt in RAP, a commercial rejuvenator was chosen, and its performance index is shown in Table 4. The rejuvenator was applied in two proportions to the mixture after RAP material and virgin aggregate have been mixed.

### 2.2. Test Methods

This sub-section explains the specimens’ fabrication and the conditioning techniques used to analyze ITZ of asphalt mixtures. Samples were divided into virgin mixture, recycled mixtures, and aged mixture, with varying content of rejuvenator, between 4% and 6%. Thin-section sample preparation was performed for structural interface observation using Nikon microscopy and a scanning electron microscope. Pull-off, SCB, and wheel track tests represent the mechanical properties of asphalt mixtures for the three groups under the same processing parameters: a 150 °C mixing temperature and 270 s mixing time. The degree of blending is mostly influenced by the mixing temperature and mixing time, according to previous research [41].

#### 2.2.1. Fluorescence Microscope Analysis

Microscope examination of a material’s surface usually requires a flat surface with extremely low roughness to achieve exact image findings [42,43]. Asphalt mixture, a porous solid substance comprising hard and soft components, cannot be employed directly for a microscopic surface evaluation [44,45]. The imaging efficiency will be greatly reduced due to the uneven surface structure of asphalt mixture [17]. As a result, the thin-section preparation procedure was presented and adopted to create a flat surface on the asphalt mixture for precise imaging characterization. The same thin-section preparation method from Xiao et al. [17] was used in this study to obtain thin sections of recycled asphalt mixture for micro-morphology study.

#### 2.2.2. Interface Strength in the Mixture

This observation can explain the compaction power, binder coating, and appropriate design of an asphalt mixture. The higher pull-off resistance at the interface of a random region is important, as service life can be accurately predicted. One type of pavement failure known as debonding separates a surface course from the base material, and this failure often appears unexpectedly and suddenly. Debonding areas with a high degree of severity might also threaten human safety. Various factors may cause debonding, including traffic-related factors (e.g., braking, stopping, accelerating, and frequent vehicle turns). Poor compaction bond, unstable mixture, incorrect choice or absence of tack coat, inappropriate compaction, and moisture damage are pavement-related causes. Pavement is more prone to wear away due to cyclic stresses where vehicles impart horizontal pressures to the pavement, such as junctions, steep ramps, and short radius bends [46]. Among the most important factors contributing to debonding are:

1. A lack of interface bond.
2. Cracks connecting the pavement’s surface to the interface.

Asphalt pavements were built with the assumption of a complete bond at asphalt interfaces; these interfaces do not exist at the time of design. As a result, understanding the state of a pavement interface may be more significant than knowing the pavement’s thickness design when choosing pavement restoration or preservation. For instance, a pavement prone to cracking due to a weak interface bonding is unsuitable for pavement preservation treatments such as seal coat, micro surfacing, or fog seal. Along with preserving pavement, it may be used for quality control and quality assurance on tack coat treatments.

**Figure 3:**
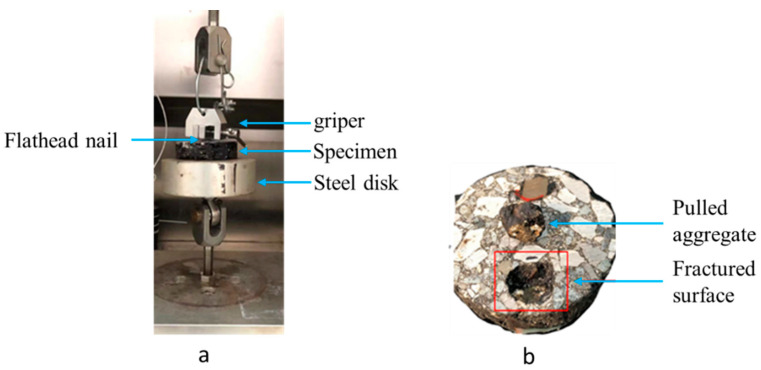
Pull-off test (**a**)Test set-up (**b**) Specimen after test.

A self-designed pull-off system was used to measure bonding strength in asphalt mixtures by applying a tensile force using Universal testing machine 130. As shown in Figure 3, a pull-off setup and the specific procedure for the test after asphalt mixture preparation are as follows:

➢ Specimens were made in Marshall form and sliced to a thickness of 20 mm, for appropriate gluing on steel disk.
➢ Specimens were cleaned with a cloth and a vacuum to eliminate any dirt and dust from the cutting process with as little force as possible, and the surface were kept dry.
➢ Preparation and application of glue substance: 0.8 ounces of glue substance was prepared according to manufacturer’s instructions. The prepared glue substance was applied on the surface of the specimen.
➢ The steel disk was gently pressed down into the top of the glue to achieve good adhesion between the specimen and the steel disk. A flathead nail was glued at the opposite side on a single aggregate; the glue should not run down the side of the test specimen when pressing down the steel disk and the flathead nail.
➢ Before testing, the specimen was allowed to cure for about 12 h at room temperature.
➢ Pull-off test: the tensile load was applied at a rate of 0.5 mm per minute [47], once the testing equipment has been properly mounted to the steel disk and the flathead nail has been gripped with a gripper. The failure modes (a) failure in the substrate, (b) failure at the interface, which is the area of concern, and (c) failure at glue were recorded.

##### Fracture Area Capture and Processing

**Figure 4:**
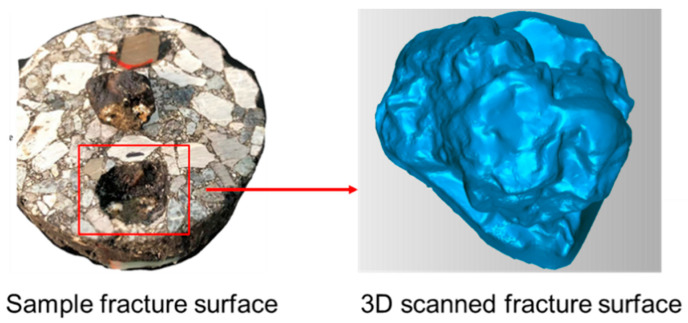
Pull-off test failure surface.

As shown in Figure 4, the area of failure is not rectangular. To obtain the actual area, a capturing protocol was initiated using HE3D 3D Scanner made by Hoyi Sunway Ltd., Yongqing, Langfang, China. The scanner allows for measuring different objects within a region of space, and acquisition of the fractured area was achieved using the toolbox Geomagic Studio 2012.1 for Windows. The scanning region is configured to acquire the fracture surface of an asphalt mixture with as many points as possible. The solid acquisition conserves the total lengths of the element in millimeters.

##### 3D Surface Area Failure Calculation

**Figure 5:**
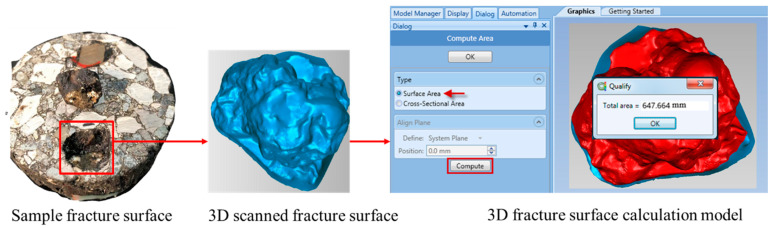
Failure surface area calculation process using Geomatic Studio toolbox.

The ideal pull-off test was designed as an alternative for measuring the interface strength that may be needed to extract an aggregate from an asphalt mixture using a tensile force. With different interpretations, the fracture area is described in 3D form, and surface area calculation steps are shown in Figure 5. On the other hand, irregularities and a non-uniform failure surface are found in fracture of asphalt mixture. During the pull-off test, aggregate selection was made according to asphalt binder penetration test standards. To estimate the genuine failure zone for a set of asphalt mixtures with different qualities, this study suggests a process that uses 3D scanning equipment.

Test measurement was analyzed as a force against displacement curve (FvsDC), and differentiation of sections within the curve will be presented in the results section. Loading speed (0.5 mm/min) and execution temperature (25 °C) are specified, and FvsDC is separated into four parts: (1) crack is not apparent, (2) crack starts to be obvious, (3) crack propagates rapidly, and (4) specimen separation. The four phases occur throughout different points in the curve generated during the pull-off test process and are described in Table 5.

##### Calculation of the Interface Strength

The total traction force on a single aggregate in an asphalt mixture is measured during the separation process. It is used to calculate the interface strength or bonding power of the asphalt mixture, and fracture surface area is obtained using a 3D scanner and toolbox Geomagic Studio 2012.1 for Windows. Certain asphalt pavement distresses start with aggregate separation from the binder. Equation (1) is used to calculate the interface bonding strength, as material failure was achieved by tensile force. The pull-off strength was defined as the highest interface bonding strength under continuous strain, and the unit for interface bonding strength is psi.

(1) $$\sigma_{ad}=F_{traction}/A$$

where $\sigma_{ad}$ is interface bonding strength; $F_{traction}$ is total traction force; and A is the interface contact area.

The traction effect of an aggregate when it separates from the mixture makes the asphalt stretch, and due to the solid interface force between asphalt molecules, there is little discernible deformation or damage when the aggregate constituents separate further. As the separation distance rose, the interface bonding strength steadily reduced until zero at the total separation point, suggesting a diminished interaction between asphalt mixture components. Some asphalt binder molecules were absorbed into some of the aggregate substrates, while some are totally separated; thus, the failure at the asphalt-aggregate interface system may be classified as an adhesive failure.

#### 2.2.3. SCB Test

SCB test specimens were prepared from Marshall samples for fracture behavior analysis and the test procedure uses ASTM D8044-2023 [48]. Firstly, the samples were cut into a 57 mm wide disk using an ordinary cutting machine. Next, the 57 mm wide disk was cut into halves using an ordinary cutting machine; each Marshall sample gave four semi-circular specimens. The testing of asphalt mixture specimens were conducted using a Universal testing machine 130, the span length for the specimens was 80 mm, with a notch depth of 10 mm, a loading rate of 0.5 mm/min until total failure (strength of 0 kPa), and a temperature condition of −10 °C. The test was run in deformation control mode at a rate of 0.5 mm/min.

#### 2.2.4. Wheel Track Test

The findings of the wheel track tests were utilized to assess rutting resistance at high temperatures [49,50]. The test process followed the ASTM D8292-2020 standard [51]. Rut plate specimens were obtained using the wheel mill, and then the wheel tracking test was performed. The test temperature was 60 °C and wheel pressure was 0.7 MPa, the round-trip rolling speed was 42 times/mm, and the test time was 1 h [52]. Finally, dynamic stability (DS) was determined to quantify the rutting deformation resistance.

## 3. Results and Discussion

### 3.1. Microscopic Morphologies

#### 3.1.1. Nikon Microscopy

**Figure 6:**
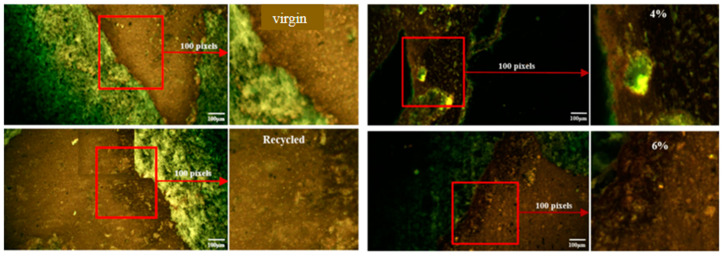
Interface morphologies of asphalt mixtures groups.

Figure 6 shows hot asphalt mixture groups (virgin, recycled, and aged with rejuvenator dosage in different proportions) under the same mixing temperature, and mixing time. From fluorescence images of virgin asphalt mixture, aggregates with varying particles sizes are in green, while the asphalt binder is the light brown region with a clear interface. The aged asphalt is dark brown, and the light-colored virgin asphalt wraps around it to form a virgin and recycled asphalt interface. This suggests that aged asphalt is difficult to soften and mix with virgin asphalt during the hot mixing process. The hot recycled asphalt mixture’s performance is poor, as the surface structure’s interface becomes weaker. It can be seen from fluorescence images that aged mixture with increasing rejuvenator material becomes fuzzy at the asphalt interface. The rejuvenator-enhanced fusion status of aged asphalt, and different sensitivities to rejuvenator, exist in aged mixtures with varying dosages. The interface of 4% rejuvenator dosage in aged asphalt mixture displays visible modifications. It will adhere to the basalt surface once 6% of rejuvenator is added, and the asphalt layer’s thickness has been lowered from tens of micrometers to a few micrometers. Aged asphalt interfaces become blurred when the rejuvenator dosage was increased to 6%, and layering of interface color from dark to light demonstrates that asphalt with a higher rejuvenator dosage is easier to merge. Y. Xiao et al. [18] investigated SBS morphologies in asphalt binders using fluorescence technology and identified a relationship between SBS morphologies and viscoelastic binder properties. Sun et al. [53] used fluorescence microscopy to analyze chemo-rheological and morphological evolution of numerous aged asphalts, finding that the essential roles of polymer breakdown and asphalt oxidation varied depending on aging stage.

**Figure 7:**
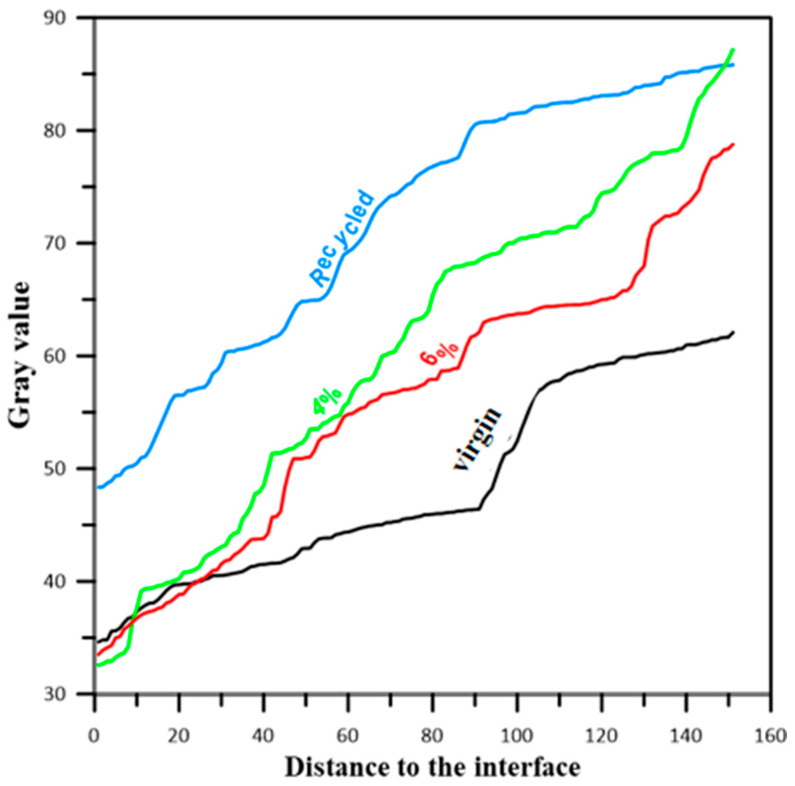
Grayscale curve of asphalt mixture groups.

The grayscale values of the interface region in asphalt mixture groups are shown in Figure 7. Grayscale curves of all samples overlap in the front section and changes in a group’s parameters will not affect the location of the interface. As depth increases, the grayscale value response of different interface structures is different, and the gray curve begins to fork, climb the “steep slope” at different inflection points, and finally stabilize through the change in the grayscale curve. It can be found that the length and slope of the “steep slope” of different interface structures are different, and the longer the steep slope, the thicker the diffusion layer between components in the asphalt mixture, and the greater the gradient, which indicates melting between the recycled mixture and the diffusion of rejuvenator in the aged mixture. The worst groups are recycled, and 4% rejuvenator dosage, for which the sample “steep slope” of the grayscale curve becomes more extended, and the slope becomes more significant, which makes the asphalt direction of mixture flow dynamic. The diffusion of aged asphalt and 4% rejuvenator slows down, hindering the blending of components between asphalt mixtures. Overall, recycled and 4% rejuvenator groups are the worst, while virgin and 6% rejuvenator groups are the best. The method played a specific auxiliary role in interface evaluation.

**Figure 8:**
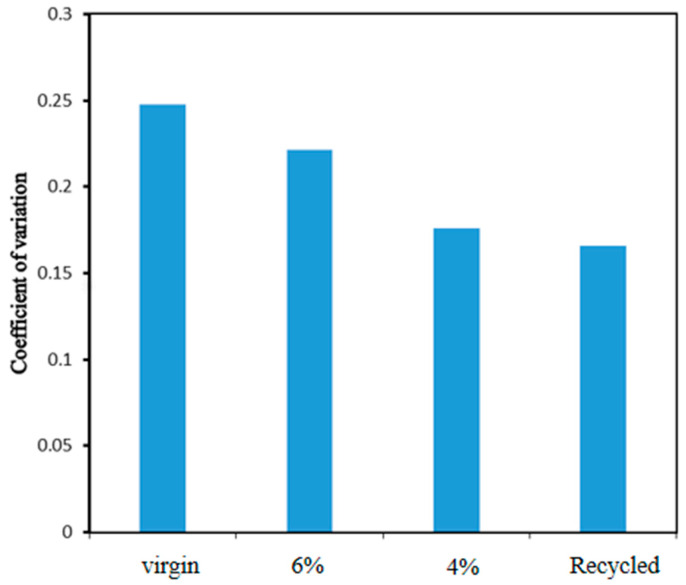
Grayscale coefficient of variation of asphalt mixture groups.

The grayscale coefficients of variation of asphalt mixture groups are shown in Figure 8. Using the same processing parameters (mixing time and mixing temperature), asphalt mixture groups’ grayscale coefficients of variation vary. By changing the mixture type, the coefficient variation of virgin mixture is higher, which indicates that the degree of component blending leads to less interface visibility. Therefore, the higher the value, the better the interface bonding, which guarantees good performance.

#### 3.1.2. Scanning Electron Microscopy

**Figure 9:**
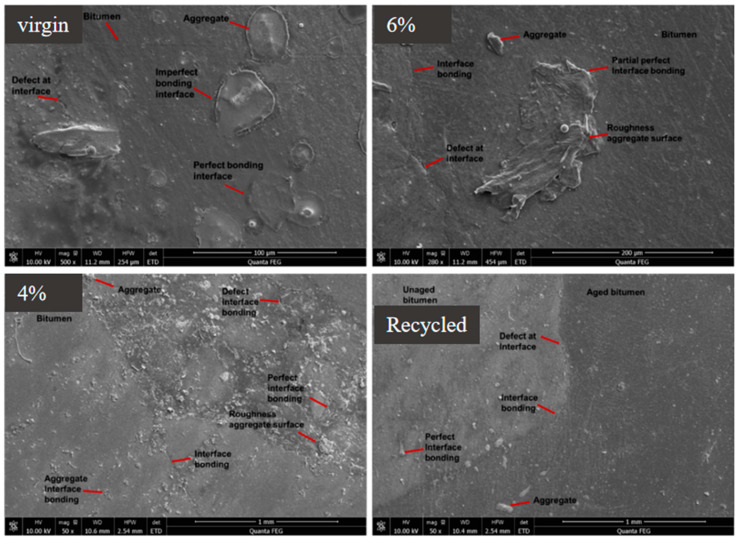
Interfacial transition zone of asphalt mixture groups at SE mode.

In this study, SEM analyses of asphalt mixture group specimens were carried out to identify interface positions or the interactions of asphalt mixture constituents. Liu et al. use SEM to observe the microstructure of aggregate to asphalt binder interfaces, the chemical interactions between asphalt and aggregate after aggregate was treated, and ITZ identification between asphalt droplets and aggregates by observation of the crystalline product [54]. Figure 9 shows SEM microscopic and relevant interface zones in asphalt mixture groups; the digital images are in different magnification. The virgin mixture was analyzed at 500×, and presented virgin asphalt and aggregate, distress, and partial or perfect bonding at interface. The 6% rejuvenator dosage mixture was at 280× and showed the regenerant distribution map and indicated the location of interface and bonding zone conditions. The asphalt mixture of 4% rejuvenator was analyzed at 50× and presented the distribution map of rejuvenator and indicated all interface conditions. The recycled asphalt mixture was analyzed at 50×, illustrating the blending state of virgin and RAP binder. Different interfaces can be seen from the RAP binder film, which is not blended with virgin binder. In mixtures with high RAP, a portion of the aged binder covers the RAP aggregate in its natural state. It adversely affects the asphalt mastic’s homogeneity, which impairs the recycled mixture’s ability to fracture.

### 3.2. Mechanical Properties

Aggregate and asphalt in an asphalt mixture are connected via an interface, also known as interface bonding. The asphalt mixture’s adhesion features at the interface zone primarily influence its mechanical qualities. Mechanical interface evaluation of different asphalt mixture groups was quantified by pull-off, SCB, and wheel track tests, and test results are shown in Table 6. Subsequently linkage between micro-morphologies and mechanical method ITZ evaluation were determined and studied.

#### 3.2.1. Pull-Off

**Figure 10:**
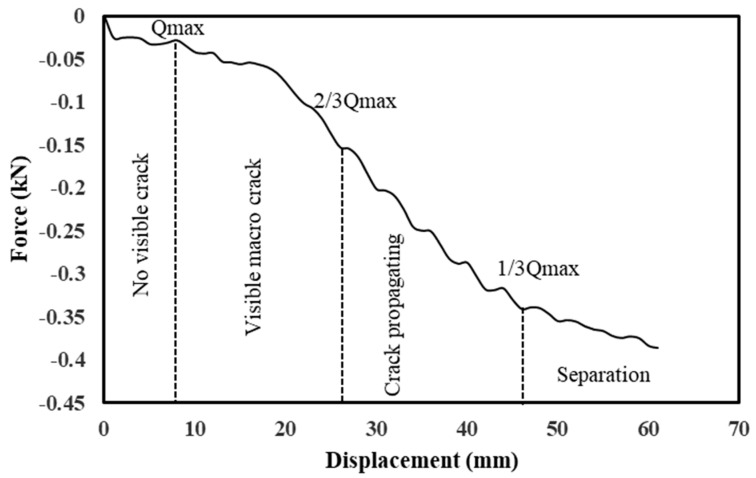
Typical pull-off test curve showing different bonding strengths stages.

**Figure 11:**
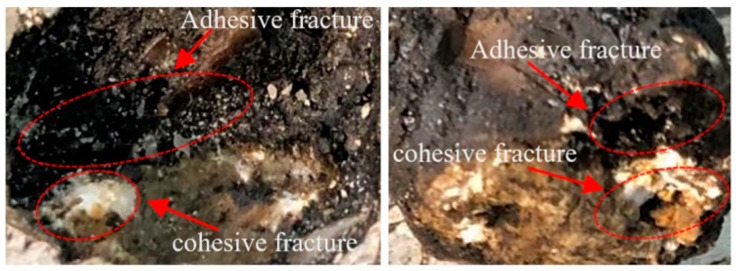
Pull-off fracture surface.

Previous investigations have shown that the chemistry and mineralogy of minerals have a significant impact on aggregate-asphalt bonding and debonding [55]. Herein, the test results of the pull-off measure interface strength of asphalt mixture are shown. The asphalt mixture groups’ bonding strength stages have been estimated, as shown in Figure 10. The pull-off test demonstrated its usefulness in measuring interface bonding conditions with both weak and strong settings, and findings indicate a significant difference in bonding strength between the asphalt mixture groups. Figure 11 shows how an asphalt mixture’s surface looks after an aggregate pull-off at room temperature with a 0.5 mm/min strain rate until ultimate failure; adhesive and cohesive fractures occurred in most of the sample’s groups. Due to the excellent mechanical qualities of basalt, failure typically occurs at the interface between aggregate and asphalt, rather than within the aggregate itself. Consequently, during direct tensile operation, there are few aggregate ruptures.

#### 3.2.2. Three-Dimensional Fracture Surface Area Acquisition and Its Interface Strength Calculation

**Figure 12:**
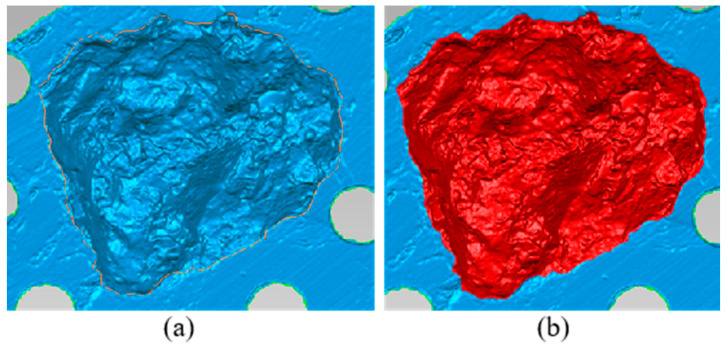
Fracture surface from 3D scanner (**a**) area acquisition, (**b**) measured area.

**Figure 13:**
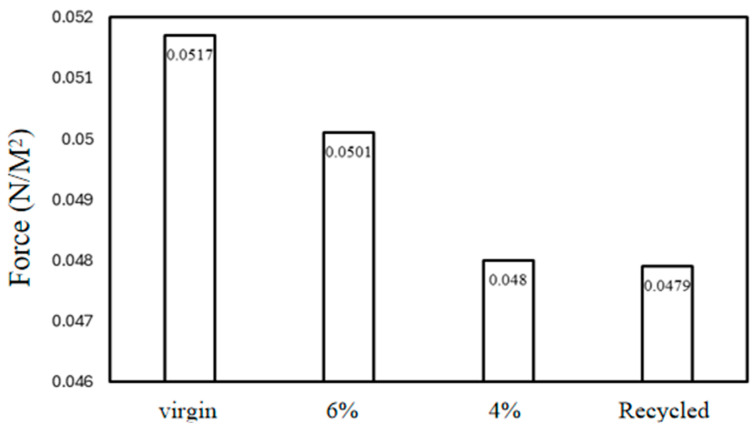
Interface bonding strength.

3D fracture surface area measurement is one of the parameters that can be used to calculate actual interface bonding strength. The strength required to pull off an aggregate from a mixture signifies the mechanical stability of the asphalt pavement. Figure 12 shows specimen fracture surface in 3D form after the pull-off test and Figure 13 shows the interface bonding strength of asphalt mixture groups, with a clear variation in bonding strength. This is attributed to operating conditions that affect adhesion, such as condition of the materials and foreign contamination. The virgin mixture demonstrated better debonding energy, as the mixture was free from any detrimental impact. The aged mixture with 6% rejuvenator dosage exhibited good performance also, even though aging has an impact on interface interaction, which influences the performance of asphalt mixtures and pavements; thus, the rejuvenator restores the durability and fracture resistance of asphalt mixture. The 4% rejuvenator dosage and recycled mixture groups have the least bonding energy among the asphalt mixture groups, the reason being inadequate restoration of asphalt mixture properties in the 4% rejuvenator dosage and mixture conditions due to the presence of RAP in the recycled mixture. Considering that interface properties control fracture mode and fracture strength, the 3D fracture surface area mode used to acquire calculation parameters provides more accuracy, as all fracture points are captured and measured by toolbox Geomagic Studio 2012.1 for Windows.

#### 3.2.3. SCB and Dynamic Stability

SCB and dynamic stability tests were used to evaluate the interface zone of asphalt mixture groups, and the test results are shown in Table 6. The asphalt mixture groups’ (recycled and aged with 4% rejuvenator dosage) stiffness modulus grows while the tensile strength falls, implying that the deeper the asphalt aging, the poorer the mixture’s low-temperature fracture resistance becomes. While the dynamic stability changes in a comparable way, the 4% rejuvenator group rose higher than all the remaining mixture types. This showed that the high-temperature stability of recycled asphalt mixed with virgin asphalt would be improved under constant mixing temperature and mixing time.

Interface studies correlate with low-temperature performance of an asphalt mixture within a particular range, according to data in Table 6. The virgin mixture’s failure strain was higher than other mixture groups at the same mixing temperature, but it demonstrated good low-temperature performance. On the other hand, improving the recycled mixture mixing process and adding an appropriate dosage of rejuvenator can increase the dynamic stability of the asphalt mixture. When the mixing procedure is poor, the viscosity of the aged asphalt will be higher. Therefore, the amount of asphalt transferred to the surface of virgin asphalt and new aggregate is reduced, making the internal high-temperature performance of the mixture poor. The weak areas lack proper blending of aged and virgin asphalt, which affects the overall anti-rutting performance of the asphalt mixture. Moreover, rejuvenator reagents can improve the high-temperature performance to a limited extent. The overall resulting difference is insignificant, as changes in composition during environmental aging and the introduction of rejuvenator and RAP will affect related properties of asphalt mixture to some extent when the alteration is minimum; therefore, the changes in properties are minimal.

### 3.3. Correlation Between Microscopic Morphology and Mechanical Properties

From a micro perspective, aging causes some asphalt molecules to alter, resulting in changes in the chemical composition of the asphalt, causing it to behave differently under fluorescence. The evaluation of interface zones in asphalt mixtures of distinct groups is obvious: a virgin mixture has a clearer constituent interface when compared to the remaining types. As the interface between virgin and aged asphalt becomes blurred, the aging asphalt layer becomes thinner, and the grayscale curve moves upward as a whole. The grayscale value of the aging asphalt interface is susceptible to rejuvenator, and its coefficient of variation decreases rapidly, but it increases with content increase. The amplitude gradually weakened, indicating that the regeneration effect achieved by the rejuvenator is limited, and only part of the aged asphalt can be regenerated. At a proper rejuvenator dosage, virgin and aged asphalt with strong fluidity could be fully miscible, and the interface fusion state will be improved, making changes in the grayscale. The thickness of aged asphalt film without adding rejuvenating reagent is about ten microns to a hundred microns, indicating that the fusion state of aged asphalt and virgin asphalt is poor and the transfer amount of aged asphalt to virgin aggregate and virgin asphalt is minimal, according to the fluorescence images. The commercial rejuvenator is composed of aromatic hydrocarbons, which can reduce the viscosity of aged asphalt and supplement light oil. As the interface between virgin and aged asphalt becomes blurred, the aging asphalt layer becomes thinner and the grayscale curve moves upward as a whole.

The fusion conditions of aged and virgin asphalt will improve the mixture’s high-temperature performance initially, then decrease it. The excessive amount of external binding rejuvenating reagent leads to the asphalt mixture’s high-temperature performance fluctuating according to the relationship between the external binding rejuvenating reagent and the grayscale value. The reason for this decrease might be that the regeneration effect of the rejuvenator is affected by temperature, asphalt aging degree, and mixing mechanical force. It is difficult for the molecules to diffuse into aged asphalt, but it is easier for them to diffuse into virgin asphalt with low viscosity, making cement in mixture sticky. The degree of imbalance damages the structure’s high-temperature stability. Table 7 shows high and low temperature performance parameter relationships with the grayscale coefficient of variation.

**Figure 14:**
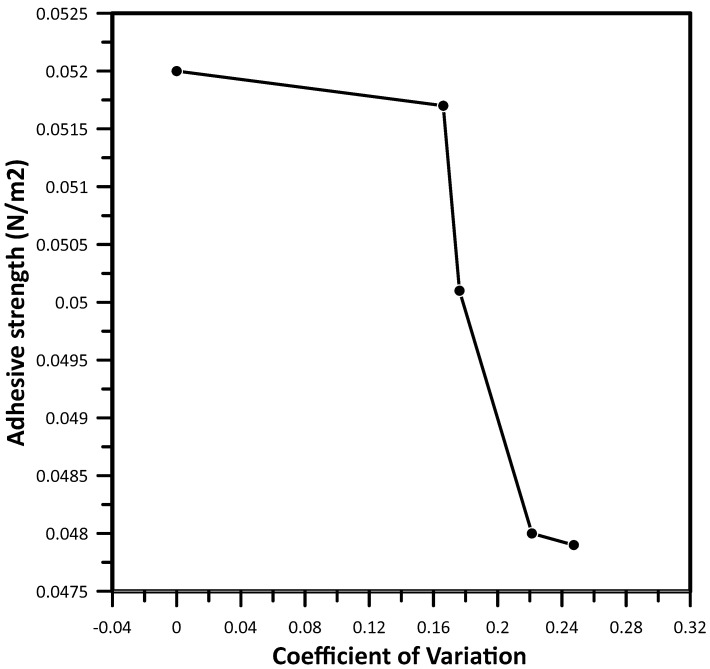
Grayscale coefficient of variations against interface bonding strength.

The asphalt mixture component interface bonding power varies as the coefficient of variation increases, as shown in Figure 14. The fusing condition of virgin mixture components has a better relationship, followed by 6% rejuvenator content mixture. The 4% rejuvenator dosage and recycled asphalt mixture interface have a poor relationship with bonding strength.

**Figure 15:**
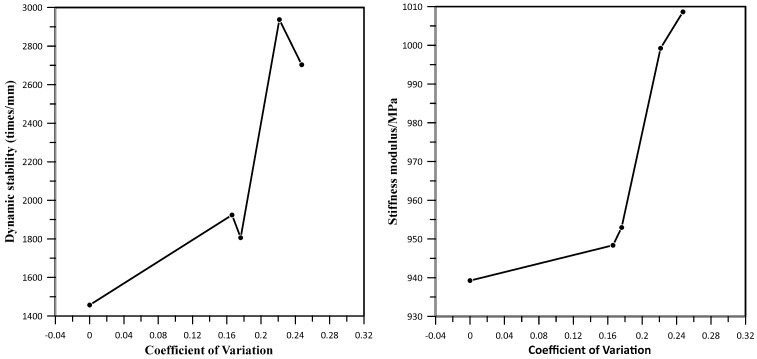
Grayscale coefficient of variations against high-temperature performance.

The dynamic stability varies up and down while the stiffness modulus rises as the coefficient of variation increases, as shown in Figure 15. The fusing conditions of virgin and aged asphalt interfaces or constituents in asphalt mixture have a poor relationship with high-temperature stability. Although fusing of aged and virgin asphalt aids dynamic stability, at high temperatures, the impact on the interface is lessened, and the recycled mixture process takes precedence in degree or dose.

**Figure 16:**
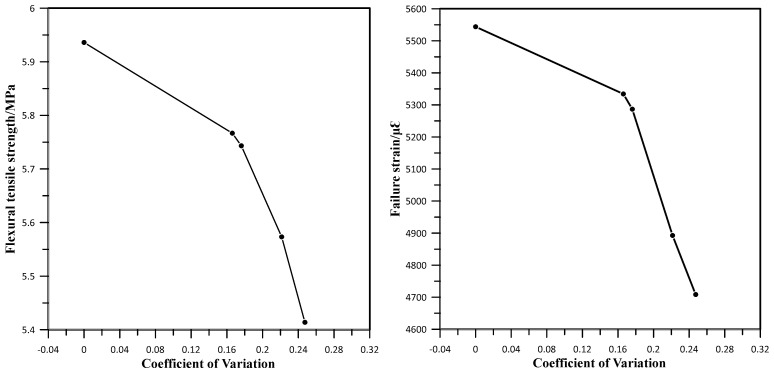
Grayscale coefficient of variations against low-temperature performance.

The relationship between a mixture’s low-temperature performance and coefficient of variation in the asphalt mixture interface is shown in Figure 16. As the degree of variation grows, the mixture’s failure strain and flexural tensile strength steadily decrease, with the virgin mixture having the highest value, followed by the 6% and 4% rejuvenator dosage asphalt mixture types, while the least was the recycled mixture group. The higher the value, the faster the rate of decrease in failure strain, suggesting that the interface in an asphalt mixture is more visible, and the components are less fused.

**Figure 17:**
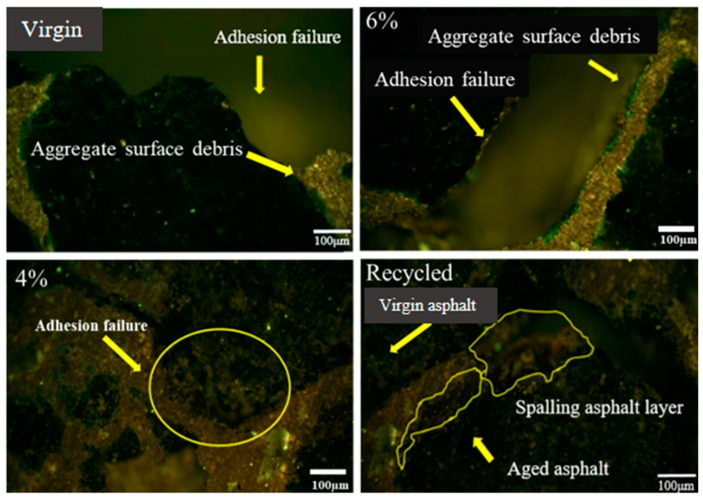
Asphalt mixture groups cracking microscopic morphology.

Microscopic morphology cracks of asphalt mixture groups are shown in Figure 17. On the surface, there are common cracking situations where fractures spread to the boundary of asphalt mixture constituents. The increasing crack pattern on interfaces between mixture components, rejuvenator or aged asphalt and virgin asphalt, which has cracked due to poor bonding, can be seen. Virgin asphalt, aggregates, and virgin asphalt coated with aged asphalt can be observed in recycled mixtures. This demonstrates that the transition between aged and virgin asphalt is relatively weak. It might be caused by a lack of interface cohesion and poor fusion between the aged and virgin asphalt. Adhesion between aged asphalt and aggregate is weak, which results in cracking of both outer (in contact with virgin asphalt) and inner (in contact with aggregate) layers of aged asphalt. The microscopic morphology of virgin asphalt mixture cracking was also presented; cracking is mostly due to adhesion failure as it spreads to the proximity of asphalt coating. After adhesion failure, basalt aggregate was exposed. The surface layer of basalt aggregate is separated from the main body, and the thickness of the aggregate outer layer adhering to asphalt is in the tens to hundreds of microns. Cracks of a typical asphalt mixture grow along the asphalt film and aggregate interface, and the aggregate surface layer is loosely structured.

Moreover, the internal action is weak, and asphalt forms the most vulnerable aggregate interface. Compared to an ordinary asphalt mixture, the interface between aged asphalt and aggregate in mixture is also separated, but the weak area lies at the interface between the virgin and aged asphalt. Mixing of aged and virgin asphalt was insufficient, making the difference between the component interfaces on both sides large and low. The volume shrinkage of the two asphalt layers is different at high temperatures, which generates more internal stress, causing cracks along the interface of the virgin and aged asphalt. Therefore, an aging asphalt layer is exposed which produces peeling. The interface between the virgin and aged asphalt is better than that between the asphalt and aggregates, but it is more fragile, so the poor fusion state will seriously reduce the low-temperature crack resistance of the asphalt mixture.

## 4. Conclusions

All results derived from the study are predicated upon unique methodologies, using specific materials, and conducted under specific research settings. Consequently, many of these conclusions cannot be extrapolated or generalized.

➢ By using two major microscopic and mechanical methods to evaluate interface zones in asphalt mixtures, various parameters that are useful in assessing asphalt pavement were derived. The interface gray curve in an asphalt mixture was obtained by using image processing software and using its gray-scale coefficient of variation to evaluate the fusion state of components in mixture. Based on asphalt’s fusing state, it was discovered that aged asphalt has a darker color under a fluorescence microscope, showing a visible interface between virgin and aged asphalt.
➢ The fusion state of the composite materials substantially corresponds with the mixture’s low-temperature anti-cracking performance. The asphalt mixture with 4% rejuvenator content and recycled mixture’s interface bonding strength and failure strain diminish as the gray variation coefficient increases; the greater the value, the faster the decreasing rate of the mixture’s failure strain.
➢ From the rutting test, the 4% rejuvenator content and recycled mixture outperforms the 6% rejuvenator content and virgin asphalt mixture group in high-temperature performance. The presence of aged asphalt in mixture and reduction in rejuvenator dosage both improve the mixture’s high-temperature performance to variable degrees. When the rejuvenator content increases, the mixture’s high-temperature performance initially increases and then falls. SCB strength test findings show that low-temperature performance of the 4% and recycled mixtures are weak compared to the 6% and virgin asphalt mixture groups.
➢ Since interactions take place at atomistic molecular scales, it is difficult to determine how and what structures and components influence the mechanical characteristics of asphalt binder and mixtures. With pull-off and its corresponding parameters (3D surface area calculation, interface bonding with fracture image), an appropriate setting is provided to evaluate asphalt mixture interfaces and offer a new perspective on deformation and failure behaviors that can be brought about.

## Figures and Tables

**Table 1:** Properties of the basalt aggregate that was utilized.

Index	Unit	Technical Standard	Test Results	Experiment Method
Stone crushing value	%	≤26	12.3	T0316
Aggregate Impact Value	%	_	5.10	BS 812–112
Los Angeles abrasion loss	%	≤28	9.0	T0317
Uniaxial Compressive Strength	MPa	_	144	TS EN 1926
Apparent density	g/cm^3^	≥2.6	3.01	T0304
Water absorption	%	≤2.0	0.7	T0304
Loss of ignition	%	_	3.42	_
SiO_2_	%	_	50.57	_
Al_2_O_3_	%	_	16.12	_
CaO	%	_	9.76	_
Fe_2_O_3_	%	_	9.02	_
MgO	%	_	4.12	_
Na_2_O	%	_	2.34	_
K_2_O	%	_	2.27	_

**Table 2:** Properties of asphalt binder.

Physical and Chemical Properties	Test Results	Requirements	Specifications
Penetration (25 °C, 100 g, 5 s), mm	69	60~80	T0604-2011
Softening point, °C	46	≥44	T0606-2011
Ductility (5 cm/min, 15 °C), cm	>100	≥40	T0605-2011
Viscosity (135 °C), Pa·s	284.5	–	T0625-2011
Saturate (%)	15.7	_	_
Aromatic (%)	31.3	_	_
Resins (%)	41.8	_	_
Asphaltenes (%)	11.2	_	_

**Table 3:** Properties of the used Mineral powder.

Index	Unit	Technical Standard	Test Results	Experiment Method
Apparent relative density	_	≥2.50	2.765	T0352
Water content	%	≤1	0.27	T0103 Drying
Granularity range <0.15 mm	%	90~100	94.6	T0351
Granularity range <0.075 mm	%	75~100	85.7	T0351
Hydrophilic coefficient	_	<1	0.765	T0353
Plasticity Index	_	<4	3	T0354
Loss of ignition	%	_	42.90	_
SiO_2_	%	_	1.72	_
Al_2_O_3_	%	_	0.82	_
CaO	%	_	52.50	_
Fe_2_O_3_	%	_	0.15	_
MgO	%	_	1.63	_
Na_2_O	%	_	0.03	_
K_2_O	%	_	0.08	_

**Table 4:** Properties of rejuvenator.

Index		Values	Requirement	Test Method
Viscosity (60 °C)/(10^−3^ Pa·s)		363.2	176~900	-
Flash point (°C)		271	≥220	-
Rate of viscosity change before and after TFOT (%)		2.60	≤3	-
Rate of viscosity change before and after TFOT (%)		−3.32	≤4, ≥−4	-
Carbon type analysis	CA%	13–16	-	D-2140
	CN%	35–38	-	D-2141
	CP%	44–46	-	D-2142
Density (g·cm^−3^)		1.012	-	D-1298

**Table 5:** Stages specimen undergoes during pull-off.

Region	Phase	Range	A Region in the Test Sample
Pre-peak load	1	0 to $\frac{1}{3}Qmax$ Start of load increase	No visible crack
	2	$\frac{1}{3}Qmax\;\mathrm{to}\;\frac{2}{3}Qmax$ Accelerated load increasing	
	3	$\frac{2}{3}Qmax\;\mathrm{to}\;Qmax$ Decreased rate of load increase	
Peak load	4	Qmax. peak load point	
Post peak load	5	$Qmax\;\mathrm{to}\;\frac{2}{3}Qmax$ Load decreasing	Starting to see a visible crack macro crack
	6	$\frac{2}{3}Qmax\;\mathrm{to}\;\frac{1}{3}Qmax$ Load decreasing	Crack propagating quickly
	7	$\frac{1}{3}Qmax\;\mathrm{to}\;0$ Load decreasing	Specimen separation

**Table 6:** SCB and dynamic stability test results of different asphalt mixture groups.

Mixture Type	Stiffness Modulus/MPa	Failure Strain/μƐ	Tensile Strength/MPa	Dynamic Stability (Times/mm)
Virgin	948.4	5334.0	5.77	1924
6% rejuvenator dosage	953.0	5286.6	5.74	1806
Recycled	1008.6	4708.4	5.41	2703.20
4% rejuvenator dosage	999.2	4892.6	5.57	2957.33

**Table 7:** Grayscale coefficient of variation relationship with high and low temperature performance.

Coefficient of Variation	Interface Bonding Strength N/m^2^	Dynamic Stability (Times/mm)	Stiffness Modulus/MPa	Failure Strain/μƐ	Flexural Tensile Strength/MPa
0.0000	0.0520	1457	939.3	5543.8	5.94
0.1661	0.0517	1924	948.4	5334.1	5.77
0.1762	0.0501	1806	953.0	5286.6	5.74
0.2213	0.0480	2937	999.2	4892.6	5.57
0.2474	0.0479	2703	1008.6	4708.4	5.41

## Data Availability

The original contributions presented in the study are included in the article, further inquiries can be directed to the corresponding authors.
